# Retraction: Evaluating the effect of therapeutic stem cells on TRAIL resistant and sensitive medulloblastomas

**DOI:** 10.1371/journal.pone.0324472

**Published:** 2025-05-12

**Authors:** The PLOS One Editors

Following the publication of this article [1] concerns were raised with the results presented in Figs 2,3,S3G, and the statistical analyses reported in this study. Specifically,

- The Fig 2D UW426 + hMSC-GFP panel appears similar to the Fig 2E hMSC-S-TRAIL panel.
- Multiple unmarked splice lines were observed in the panels presented in Fig 3G and Fig S3G results.
- The Statistical Analysis section of the Materials and Methods reports that data were analysed using Student t-tests when comparing 2 groups. However, multiple experiments presented in this article appear to present more than 2 variables, suggesting that statistical tests designed for the comparison of multiple variables should have been used instead.

Regarding the panel duplication in Fig 2, the corresponding author clarified that the image representing the UW426 + hMSC-GFP condition in Fig 2D and the image representing the hMSC-GFP condition in Fig 2E represent the same mouse. They stated that Fig 2D presents the fold change in tumor growth for hMSC-GFP treated mice relative to the untreated mice control, and that Fig 2E presents fold change in tumor growth for (UW426 +) hMSC-S-TRAIL treated mice relative to the (UW426 +) hMSC-GFP treated group. In light of this explanation, the *PLOS One* Editors consider the concerns with Fig 2 resolved.

Regarding the splice line concerns with Figs 3 and S3, the corresponding author confirmed that during the preparation of the western blot results presented in the original article [1], the underlying blots were spliced to remove non-pertinent lanes. The underlying blots provided for editorial review confirm that the published figure presents panels spliced from the same underlying blots, and resolve the journal’s concerns with the results presented in Figs 3 and S3.

Regarding the concerns pertaining to the statistical analysis reported in this article [1], the corresponding author stated that the analysis reported in [1] focused specifically on comparing each treatment group to the control group independently. The corresponding author commented that they did not conduct multiple comparisons between the treatment groups themselves and therefore the use of multiple comparison correction methods were not required. They also stated that individual-level data underlying the results presented in this article are no longer available.

The statistics reported in the article and the author’s clarification of the statistical approach were reviewed by an independent statistical expert, who stated that the statistics reported in the article are inappropriate. They commented that the comparisons reported in the figures present multiple testing, and that this should have been accounted for when drawing conclusions. They also commented that the single t-test approach is totally inefficient and that a regression approach would have been more appropriate for this study. In the absence of the original data underlying the published results, these concerns cannot be fully resolved.

The *PLOS One* Editors retract this article in light of the statistical review, which calls into question the validity and reliability of the conclusions.

TBO and KS did not agree with the decision. IN, SW, and MA either did not respond directly or could not be reached.
